# Supplementary material for: A cleavable chimeric peptide with targeting and killing domains enhances LPS neutralization and antibacterial properties against multi-drug resistant E. coli
Source: Commun Biol. 2023 Nov 16;6:1170. doi: 10.1038/s42003-023-05528-0 (PMC10654507; doi:10.1038/s42003-023-05528-0)
Supplement: Supplementary file 2 — Supplementary Information [file 42003_2023_5528_MOESM2_ESM.pdf]

Supporting Information

A cleavable chimeric peptide with targeting and killing domains enhances LPS neutralization and antibacterial properties against multi-drug resistant *E. coli*

Zhenlong Wang[1,2], Da Teng[1,2], Ruoyu Mao[1,2], Ya Hao[1,2], Na Yang[1,2], Xiumin Wang[1,2*], Jianhua Wang[1,2*]

[1] *Key Laboratory of Feed Biotechnology, Ministry of Agriculture and Rural Affairs, Beijing 100081, People's Republic of China*

[2] *Gene Engineering Laboratory, Feed Research Institute, Chinese Academy of Agricultural Sciences, Beijing 100081, People's Republic of China*

* These corresponding authors jointly supervised this work

Prof., Ph.D., and PI. Jianhua Wang, and postal address of all authors as:

Gene Engineering Laboratory, Feed Research Institute

Chinese Academy of Agricultural Sciences, 12 Zhongguancun Nandajie St., Haidian District, Beijing 100081, P. R. China

E-mail address: wangjianhua@caas.cn; wangxiumin@caas.cn

Phone: 0086-10-82106081, 0086-10-82106079; Fax: 0086-10-82106079

Contents:

1. Supplementary Table 1. Percentage of secondary structures contents of L7 in different membrane environments --- **S3**
2. Supplementary Table 2. Percentage of secondary structures contents of R7 in different membrane environments --- **S3**
3. Supplementary Table 3. Fragment analysis of R7 by LC-MS in mouse serum -----**S4-S8**
4. Supplementary Table 4. Fragment analysis of R7 by LC-MS in mouse -----------------**S9**
5. Supplementary Table 5. Fragment analysis of R7 by MALDI-MS in mouse ------------**S9**
6. Supplementary Table 6. The MIC (μM) values of LBP14-RKRR, LBP14-RKR, L7 and R-L7---**S9**
7. Supplementary Table 7. top five degradation combined fragments analysis of R7 in serum --- **S9**
8. Supplementary Figure 1**.** R7 is cut by furin and detected via HPLC (A) R7, (B) LBP14-RKRR, (C) L7, (D)R7 incubated with furin for 15h. -----------------------------------**S10**
9. Supplementary Figure 2**.** Serum stability and release of peptides in human serum. --- **S11**
10. Supplementary Figure 3**.** Therapeutic efficacy of cleavable chimeric peptide R7 in the mice challenged with MDR *E. coli* or LPS--- **S12**
11. Supplementary Figure 4**.** Efficacy of different doses of cleavable chimeric peptide R7 in the mice challenged with MDR *E. coli*. --- **S12**
12. Supplementary Figure 5**.** Effects of R7 and L7 on LPS-induced NF-kB and MAPK signaling pathways in lung tissues. --- **S13**
13. Supplementary Figure 6**.** A full and uncropped presentation for Supplementary Figure 5. -- **S13**

Supplementary Table 1 CD analysis of secondary structures of L7 in water, SDS, and 50% TFE.

Secondary structures	The percentages of secondary structures (%) of L7				
	H_2O	5 mM SDS	20 mM SDS	40 mM SDS	50% TFE
α-Helix	4.8	32.0	10.2	10.1	13.9
Antiparallel	26.3	11.1	41.6	41.4	42.0
Parallel	2.6	9.2	4.0	4.0	5.1
β-Turn	25.4	17.8	19.9	19.9	18.3
Random coil	40.8	30.0	24.4	24.6	20.7

Supplementary Table 2 CD analysis of secondary structures of R7 in water, SDS, and 50% TFE.

Secondary structures	The percentages of secondary structures (%) of R7				
	H_2O	5 mM SDS	20 mM SDS	40 mM SDS	50% TFE
α-Helix	3.5	35.4	22.9	23.5	50.4
Antiparallel	10.1	8.5	26.8	25.4	0.8
Parallel	1.7	9.8	6.5	6.5	8.7
β-Turn	31.2	17.5	20.9	21.1	16.4
Random coil	53.5	28.8	23.0	23.5	23.7

Supplementary Table 3 Fragment analysis of R7 by LC-MS in mouse serum

Sequence	# PSMs	Modifications	q-Value	MH+ [Da]	RT [min]
RFKAWRWAWRMKKLAAPS	94		0	2289.27024	45.48
RVQGRWKVRASFF	67		0	1636.92698	26.82
RFKAWRWAWRmKKLAAPS	26	M11(Oxidation)	0	2305.26517	31.54
AWRWAWRMKKLAAPS	21		0	1858.00759	43.32
KRRFKAWRWAWRMKKLAAPS	15		0	2573.46988	25.30
RVQGRWKVRASFFKRKRRFKA WRWAWRMKKLAAPS	13		0	4475.58118	29.64
RFKAWRWAWRm	12	M11(Oxidation)	0.002	1609.82924	37.53
RFKAWRWAWRM	11		0	1593.83730	45.59
ASFFKRKRRFKAWRWAWRMK KLAAPS	11		0.002	3309.87905	28.34
RRFKAWRWAWRMKKLAAPS	10		0	2445.38614	29.25
RFKAWRWAW	9		0.002	1306.69306	46.55
WAWRMKKLAAPS	9		0	1444.78935	32.91
RVQGRWKVRASFFK	8		0	1765.02102	23.79
WRWAWRMKKLAAPS	8		0	1786.96682	42.45
KRRFKAWRWAWRmKKLAAPS	7	M13(Oxidation)	0.002	2589.46572	23.61
VQGRWKVRASFF	6		0.002	1480.81656	36.40
RVQGRWKVRASFFKR	6		0	1921.11692	58.53
RVQGRWKVRASFFKRKRRFKA WR	6		0	3049.77705	22.39
RVQGRWKVRASFFKRKRRFKA WRWAWR	6		0	3649.10657	26.13
RVQGRWKVRASFFKRKRRFKA WRWAWRmKKLAAPS	6	M28(Oxidation)	0	4491.56639	25.08
AWRWAWRm	5	M8(Oxidation)	0.002	1178.56327	51.62
RFKAWRWAWRMKKL	5		0.002	1963.11245	32.38
RVQGRWKVRASFFKRKR	5		0	2205.31015	20.07
ASFFKRKRRFKAWRWAWR	5		0.002	2483.40322	25.11
RVQGRWKVRASFFKRKRRFKA W	5		0	2893.67902	22.54
RFKAWRWAWRMK	4		0	1721.93479	31.94
RRFKAWRWAWRM	4		0	1749.93711	33.92
AWRWAWRMKKLAAP	4		0	1770.97646	45.52
RFKAWRWAWRMKKLA	4		0	2034.14627	33.03
ASFFKRKRRFKAWRWAW	4		0.002	2327.30190	30.69
VQGRWKVRASFFKRKRRFKA	4		0	2737.59658	23.80

W					
KRKRRFKAWRWAWRmKKLAAPS	4	M15(Oxidation)	0.002	2873.65390	22.75
ASFFKRKRRFKAWRWAWRMKKLAAP	4		0	3222.85195	30.30
WKVRASFFKRKRRFKAWRWAWRMKKLAAPS	4		0.002	3879.21430	45.43
VQGRWKVRASFFKRKRRFKAWRWAWRMKKLAAPS	4		0	4319.46241	32.84
AWRWAW	3		0.006	875.43163	62.07
KRRFKAWRWAW	3		0.002	1590.89847	26.70
RWAWRMKKLAAPS	3		0	1600.89687	26.19
VQGRWKVRASFFK	3		0	1608.92360	26.42
WKVRASFFKRKR	3		0.002	1608.95704	20.97
KRRFKAWRWAWR	3		0.002	1746.99660	24.45
VQGRWKVRASFFKR	3		0	1765.01905	23.78
KRRFKAWRWAWRM	3		0.002	1878.04624	26.45
KRRFKAWRWAWRm	3	M13(Oxidation)	0.002	1894.03028	24.54
RKRRFKAWRWAWRM	3		0	2034.13877	24.92
RVQGRWKVRASFFKRK	3		0.004	2049.20826	20.98
RFKAWRWAWRmKKLAA	3	M11(Oxidation)	0.002	2121.18558	34.45
RFKAWRWAWRMKKLAAP	3		0.002	2202.23478	42.84
FKRKRRFKAWRWAWRM	3		0.002	2309.30117	24.72
KRRFKAWRWAWRMKKLA	3		0.002	2318.33950	24.88
RRFKAWRWAWRMKKLAAP	3		0.002	2358.34302	31.87
SFFKRKRRFKAWRWAWR	3		0	2412.35854	24.96
KRRFKAWRWAWRMKKLAAP	3		0	2486.45693	26.33
RVQGRWKVRASFFKRKRRF	3		0	2508.48330	20.78
VRASFFKRKRRFKAWRWAW	3		0	2582.47573	28.46
RKRRFKAWRWAWRMKKLAAPS	3		0	2729.57517	24.78
RKRRFKAWRWAWRmKKLAAPS	3	M14(Oxidation)	0.002	2745.56989	22.72
QGRWKVRASFFKRKRRFKAWR	3		0.002	2794.62832	21.92
KRKRRFKAWRWAWRMKKLAAPS	3		0	2857.66935	24.48
RVQGRWKVRASFFKRKRRFKAWRWAW	3		0	3493.00332	29.74
RVQGRWKVRASFFKRKRRFKAWRWAWRMKKLAAP	3		0	4388.53725	30.62
RVQGRWKV	2		0.004	1028.61077	19.87
RVQGRWKVR	2		0.004	1184.71296	17.31

RVQGRWKVRA	2		0.004	1255.74868	17.90
AWRWAWRMK	2		0.002	1290.66785	41.62
VQGRWKVRASF	2		0.002	1333.75174	24.64
RVQGRWKVRAS	2		0.002	1342.78078	17.38
FKAWRWAWRM	2		0.002	1437.73440	55.52
RFKAWRWAWR	2		0	1462.78921	33.51
RVQGRWKVRASF	2		0	1489.84865	22.82
WRWAWRMKKLA	2		0.002	1531.84742	39.99
WRWAWRMKKLAA	2		0.002	1602.88112	40.48
AWRWAWRMKKLA	2		0.002	1602.88309	40.51
RRFKAWRWAWR	2		0.002	1618.90485	25.63
RRFKAWRWAWRm	2	M12(Oxidation)	0.002	1765.94175	26.09
SFFKRKRRFKAWR	2		0.002	1813.06058	19.87
AWRWAWRmKKLAAPS	2	M8(Oxidation)	0.006	1874.00066	36.29
SFFKRKRRFKAWRW	2		0.006	1999.14732	23.77
KRKRRFKAWRWAWR	2		0.002	2031.18953	21.28
RFKAWRWAWRMKKLAA	2		0.002	2105.17866	35.25
FKAWRWAWRMKKLAAPS	2		0.002	2133.17044	45.43
FKAWRWAWRmKKLAAPS	2	M10(Oxidation)	0.004	2149.16191	34.44
KRKRRFKAWRWAWRM	2		0.002	2162.23130	24.23
RFKAWRWAWRmKKLAAP	2	M11(Oxidation)	0.004	2218.24656	28.49
KRRFKAWRWAWRmKKLAA	2	M13(Oxidation)	0	2405.38144	24.78
WKVRASFFKRKRRFKAWR	2		0	2453.45011	22.87
SFFKRKRRFKAWRWAWRM	2		0.004	2543.41077	28.52
RWKVRASFFKRKRRFKAWR	2		0	2609.53870	21.00
RKRRFKAWRWAWRMKKLAAP	2		0	2642.54844	24.99
VRASFFKRKRRFKAWRWAWR	2		0	2738.57192	24.93
ASFFKRKRRFKAWRWAWRMK	2		0.002	2742.52407	25.32
KRKRRFKAWRWAWRMKKLAA P	2		0	2770.63711	24.69
VRASFFKRKRRFKAWRWAWR M	2		0	2869.61611	28.39
FKRKRRFKAWRWAWRMKKLA APS	2		0	3004.74442	24.68
FKRKRRFKAWRWAWRmKKLA APS	2	M16(Oxidation)	0.002	3020.72597	23.46
ASFFKRKRRFKAWRWAWRmK KLAA	2	M19(Oxidation)	0.002	3141.79392	26.45
SFFKRKRRFKAWRWAWRMKK LAAPS	2		0.004	3238.85219	28.08

ASFFKRKRRFKAWRWAWRmK KLAAPS	2	M19(Oxidation)	0.002	3325.87246	24.77
RASFFKRKRRFKAWRWAWRM KKLAAPS	2		0.002	3465.98554	26.29
RVQGRWKVRASFFKRKRRFKA WRWAWRM	2		0.002	3780.14737	30.18
QGRWKVRASFFKRKRRFKAWR WAWRMKKLAAPS	2		0.002	4220.39381	31.39
RVQGRWK	1		0.004	929.54248	13.45
SFFKRKR	1		0.006	968.57819	15.63
AWRWAWR	1		0.006	1031.52995	46.19
ASFFKRKR	1		0.006	1039.61652	16.92
RFKAWRWA	1		0.006	1120.62183	26.22
WAWRmKKL	1	M5(Oxidation)	0.006	1134.62590	24.05
KRRFKAWR	1		0.006	1147.69562	17.55
FKAWRWAW	1		0.006	1150.59416	56.35
AWRWAWRM	1		0.006	1162.56726	56.15
WAWRMKKLA	1		0.002	1189.66997	29.35
GRWKVRASFF	1		0.006	1253.68789	33.08
RRFKAWRWA	1		0.002	1276.71938	23.95
VRASFFKRKR	1		0.004	1294.78322	17.47
WKVRASFFKR	1		0.006	1324.76579	24.80
WAWRMKKLAAP	1		0.002	1357.75786	36.85
QGRWKVRASFF	1		0.002	1381.74626	32.97
KRRFKAWRWA	1		0.008	1404.81203	21.22
WAWRmKKLAAPS	1	M5(Oxidation)	0.006	1460.78691	25.01
FKAWRWAWRMK	1		0.006	1565.82363	42.72
VRASFFKRKRRF	1		0.008	1597.95449	19.28
RWAWRmKKLAAPS	1	M6(Oxidation)	0.002	1616.88759	23.66
WRWAWRmKKLAA	1	M7(Oxidation)	0.006	1618.87819	37.10
WRWAWRMKKLAAP	1		0.002	1699.93606	45.13
ASFFKRKRRFKAW	1		0.002	1727.99748	21.09
RFKAWRWAWRmK	1	M11(Oxidation)	0.002	1737.93516	25.45
RKRRFKAWRWAW	1		0.002	1746.99350	24.98
RFKAWRWAWRMKK	1		0.002	1850.03446	25.10
RRFKAWRWAWRMK	1		0.002	1878.03861	25.57
FKAWRWAWRMKKLA	1		0.002	1878.04319	43.08
ASFFKRKRRFKAWR	1		0.006	1884.09925	20.60
RKRRFKAWRWAWR	1		0.002	1903.09492	23.27

QGRWKVRASFFKRKR	1		0.008	1950.14162	20.31
RFKAWRWAWRmKKL	1	M11(Oxidation)	0.002	1979.11451	25.22
KAWRWAWRMKKLAAPS	1		0.002	1986.10178	32.58
KAWRWAWRmKKLAAPS	1	M9(Oxidation)	0.006	2002.10112	25.12
KRRFKAWRWAWRMK	1		0.002	2006.13172	24.55
KRRFKAWRWAWRmK	1	M13(Oxidation)	0.006	2022.13087	22.88
FKRKRRFKAWRWAW	1		0.002	2022.15799	24.72
FKAWRWAWRMKKLAAP	1		0.004	2046.13584	45.63
ASFFKRKRRFKAWRW	1		0.002	2070.18186	23.99
KRRFKAWRWAWRMKKL	1		0.002	2247.31687	24.78
WKVRASFFKRKRRFKAW	1		0.002	2297.34153	24.12
FFKRKRRFKAWRWAWR	1		0.002	2325.33730	24.42
VQGRWKVRASFFKRKRRF	1		0.006	2352.37783	21.11
KRRFKAWRWAWRMKKLAA	1		0.002	2389.38016	24.98
VRASFFKRKRRFKAWRWA	1		0.006	2396.38979	23.94
RWKVRASFFKRKRRFKAW	1		0.002	2453.44936	23.55
RRFKAWRWAWRmKKLAAPS	1	M12(Oxidation)	0.004	2461.37022	24.88
KRRFKAWRWAWRmKKLAAP	1	M13(Oxidation)	0.002	2502.43650	25.13
KRKRRFKAWRWAWRMKKL	1		0.006	2531.51319	23.75
ASFFKRKRRFKAWRWAWRM	1		0	2614.43862	29.93
ASFFKRKRRFKAWRWAWRm	1	M19(Oxidation)	0.007	2630.43198	25.30
RVQGRWKVRASFFKRKRRFK	1		0.004	2636.57697	20.05
QGRWKVRASFFKRKRRFKAW	1		0.002	2638.51992	23.57
RASFFKRKRRFKAWRWAWR	1		0.002	2639.50178	24.70
GRWKVRASFFKRKRRFKAWR	1		0.002	2666.57262	21.54
RVQGRWKVRASFFKRKRRFKA	1		0.002	2707.61121	20.74
VRASFFKRKRRFKAWRWAWR m	1	M21(Oxidation)	0.006	2885.61318	25.05
ASFFKRKRRFKAWRWAWRMK KL	1		0.002	2983.72207	26.38
WKVRASFFKRKRRFKAWRWA WR	1		0	3052.75942	29.70
ASFFKRKRRFKAWRWAWRMK KLA	1		0.004	3054.76484	26.68
FFKRKRRFKAWRWAWRMKKL AAPS	1		0.006	3151.79945	25.97
SFFKRKRRFKAWRWAWRmKK LAAPS	1	M18(Oxidation)	0.002	3254.82461	24.86
VRASFFKRKRRFKAWRWAWR MKKLAAP	1		0.006	3478.01855	28.52
VRASFFKRKRRFKAWRWAWR	1	M21(Oxidation)	0.006	3581.03603	24.90

mKKLAAPS

Supplementary Table 4 Fragment analysis of R7 by LC-MS in mouse

Sequence	# PSMs	Modifications	q-Value	MH+ [Da]	RT [min]
RMKKLAAPS	1		0	1001.59220	11.21

Supplementary Table 5 Fragment analysis of R7 by MALDI-MS in mouse [a].

Peptide	Fragment mass (Da)	Assignment
R7	4477.602	RVQGRWKVRASFFKRKRRFKAWRWAWRMKKLAAPS
	3310.804	ASFFKRKRRFKAWRWAWRMKKLAAPS
	2289.3670	RFKAWRWAWRMKKLAAPS
	1858.0977	AWRWAWRMKKLAAPS
	1637.0069	RVQGRWKVRASFF
	1480.9025	VQGRWKVRASFF[b]
		RWKVRASFFKR[b]
	1184.7919	RVQGRWKVR
	929.6156	RVQGRWK

[a] Peptides with observed intermediate fragments are listed here.

[b] These fragments share same molar mass.

The arrow indicates the location where the cleavable sites occurred at R.

Supplementary Table 6 The MIC (μM) values of LBP14-RKRR, LBP14-RKR, L7 and R-L7.

Strains	LBP14-RKRR	LBP14-RKR	L7	R-L7
Escherichia coli CVCC195	2.26	2.26	2.26	2.26
S. typhimurium CVCC533	4.53	4.53	18.1	18.1
Staphylococcus aureus ATCC43300	4.53	4.53	18.1	18.1
S. aureus CVCC546	9.06	9.06	18.1	18.1

Supplementary Table 7 top five degradation combined fragments analysis of R7 in serum [a]

R7	RVQGRWKVRASFFK-RKRR- FKAWRWAWRMKKLAAPS	
1	RVQGRWKVRASFFK-RKR	R- FKAWRWAWRMKKLAAPS
2	RVQGRWKVRASFF	K-RKRR- FKAWRWAWRMKKLAAPS
3	RVQGRWKVRASFFK-RKRR- FK	AWRWAWRMKKLAAPS
4	RVQGRWKVRASFFK-R	KRR- FKAWRWAWRMKKLAAPS
5	RVQGRWKVRASFFK-RK	RR- FKAWRWAWRMKKLAAPS

[a] Table S7 is summarized from table S3.

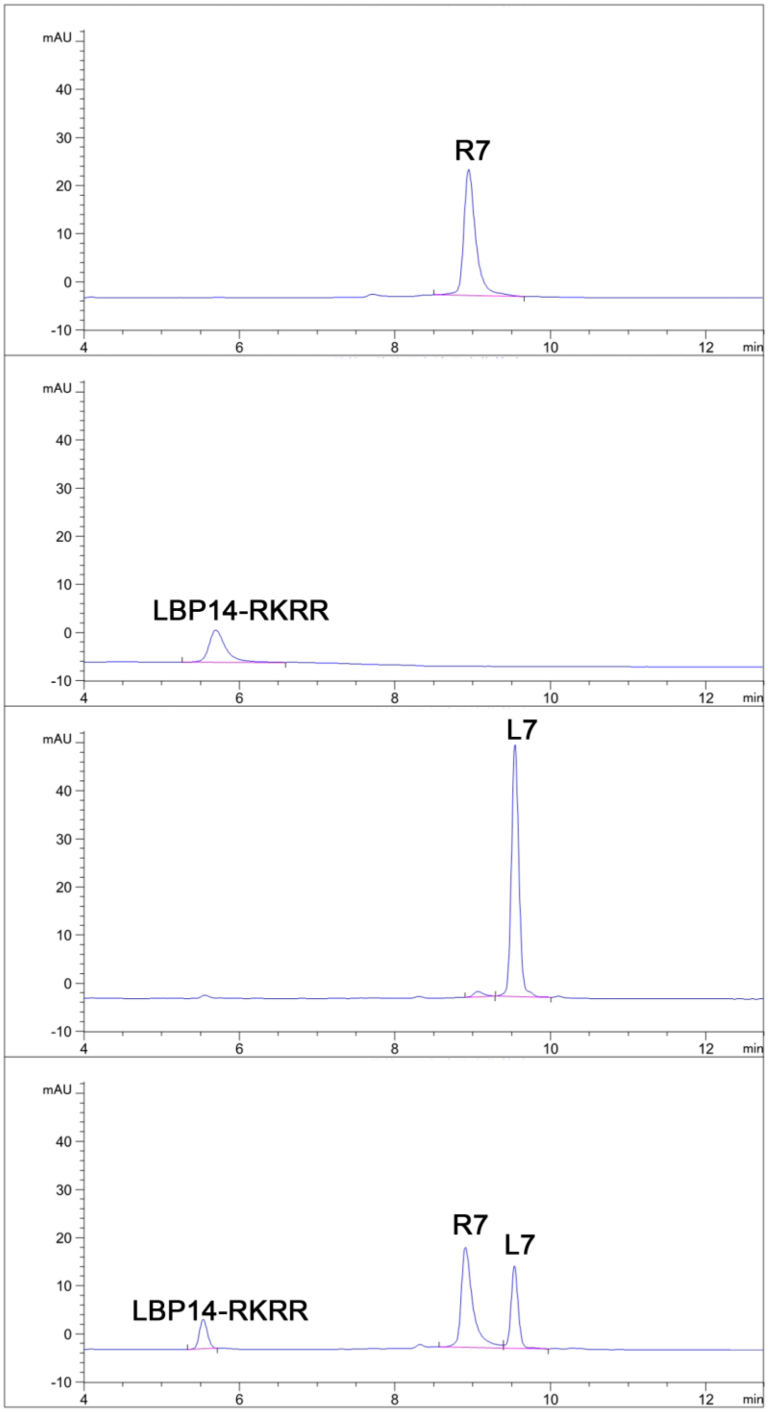

Supplementary Figure 1. R7 is cut by furin and detected via HPLC. **a** R7, **b** LBP14-RKRR, **c** L7, **d** R7 incubated with furin for 15h.

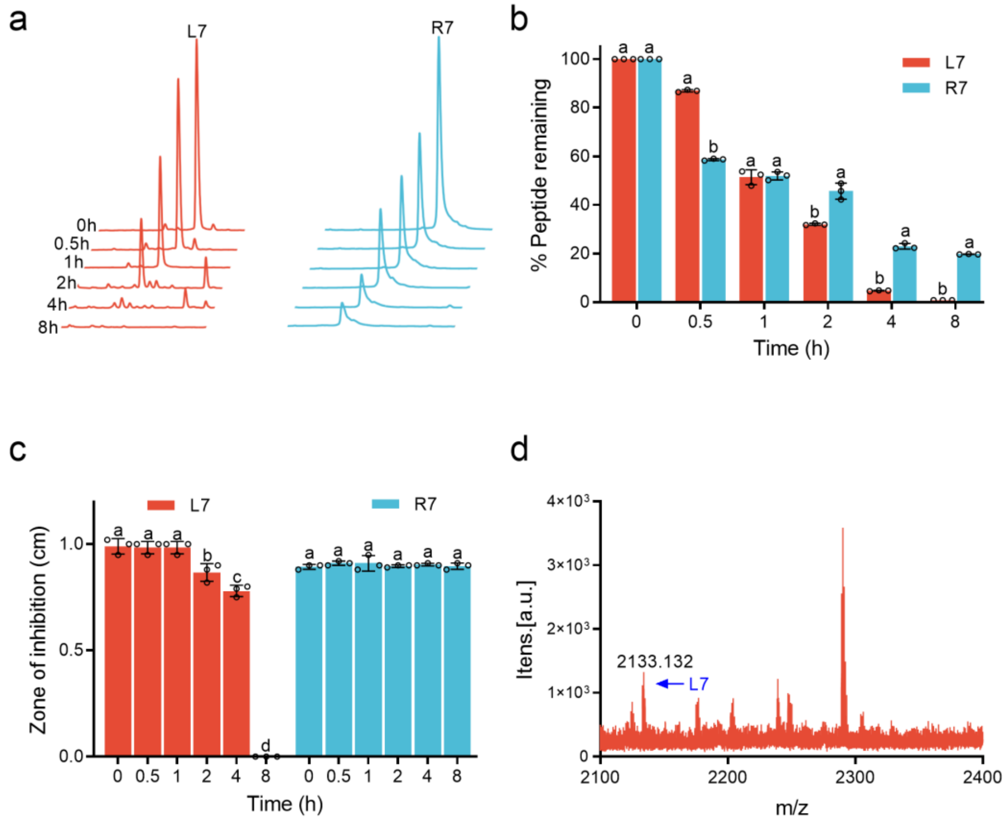

Supplementary Figure 2. Serum stability and release of peptides in human serum. a Detection of the peak area change of peptides in serum by RP-HPLC. **b** The remaining rate of peptides in bovine serum. **c** The antibacterial activity of peptides against MDR *E. coli* in serum detected by an inhibition assay. **d** The MALDI-TOF mass spectrometry of R7 after incubation in bovine serum for 2 h. The experiments were repeated three times. Data in **a** and **d** is representative of three biological replicates. The results are given as the mean ± SD of three independent experiments in **b** and **c**. Different lower-case letters indicate a significant difference between the two groups ($p<0.05$).

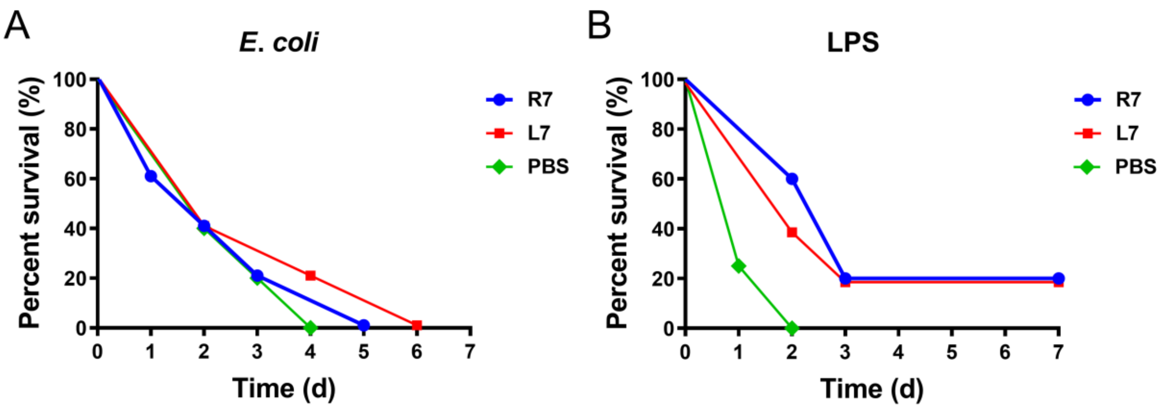

Supplementary Figure 3. Therapeutic efficacy of cleavable chimeric peptide R7 in the mice challenged with MDR *E. coli* or LPS After the intraperitoneal injection with MDR *E. coli* **(a)** or LPS **(b)** at a concentration of LD100, the mice were treated with 7 μmol/kg R7 at 0.5 h and 8 h, respectively. The mouse survival was recorded for 7 d. The mice injected with MDR E. coli/LPS or saline were used as negative or blank controls, respectively

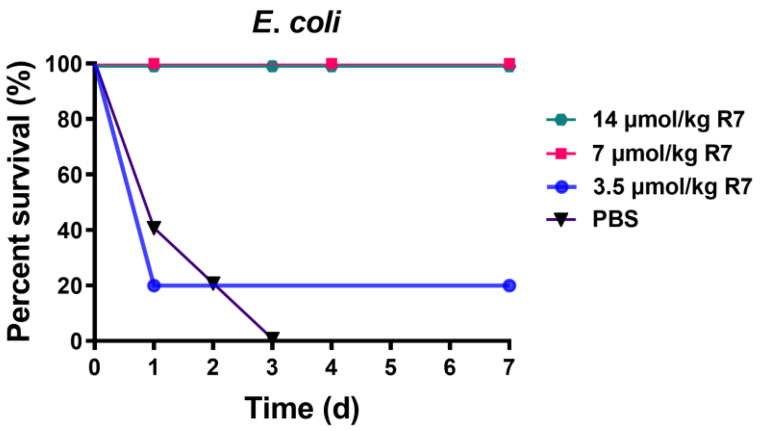

Supplementary Figure 4. Efficacy of different doses of cleavable chimeric peptide R7 in the mice challenged with MDR *E. coli*. Mice were intraperitoneally injected with 3.5, 7 or 14 μmol/kg R7 at 0 h (five mice/group), followed by injection with MDR *E. coli* CVCC195 (0.5×10^9 CFU/mouse) at 6 h. PBS was used as control. Survival was recorded for 7 d.

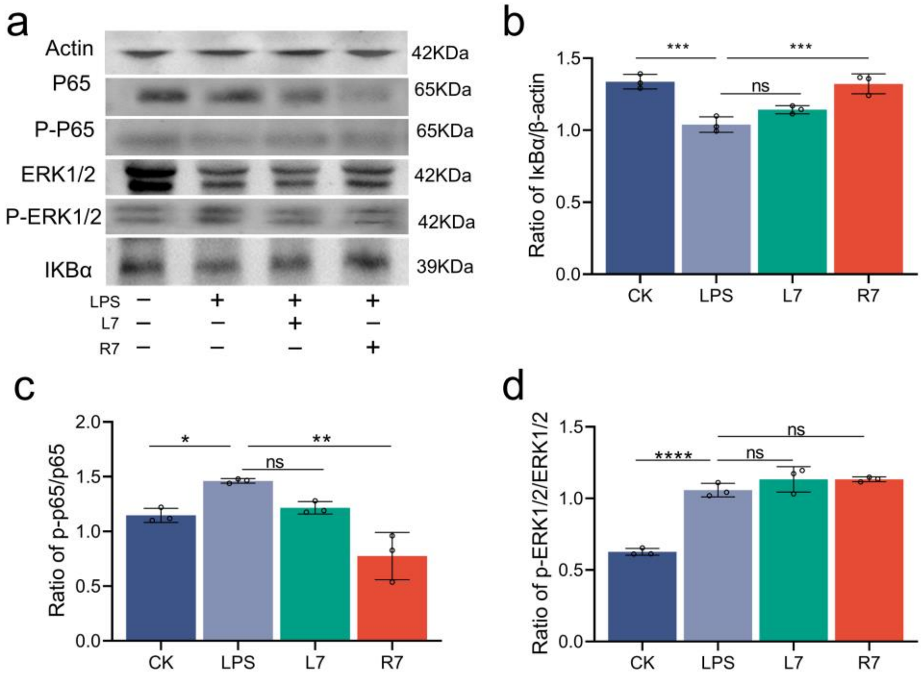

Supplementary Figure 5. Effects of R7 and L7 on LPS-induced NF-kB and MAPK signaling pathways in lung tissues. The mice were pretreated with R7 and L7, and followed by injected with LPS. **a** The protein levels of p65, p-p65, ERK1/2, p-ERK1/2, and IκBα in lungs were analyzed by western blotting. **b** Densitometric analysis of p-ERK1/2/ERK1/2 ratio. **c**. Densitometric analysis of p-p65/p65 ratio. **d** Densitometric analysis of IκBα/β-actin ratio. All data were analyzed with one-way ANOVA, and data are means ± SD (n = 3). *p*-values< 0.05 were considered significant. **, $p < 0.01$, ***, $p < 0.001$, ****, $p < 0.0001$.

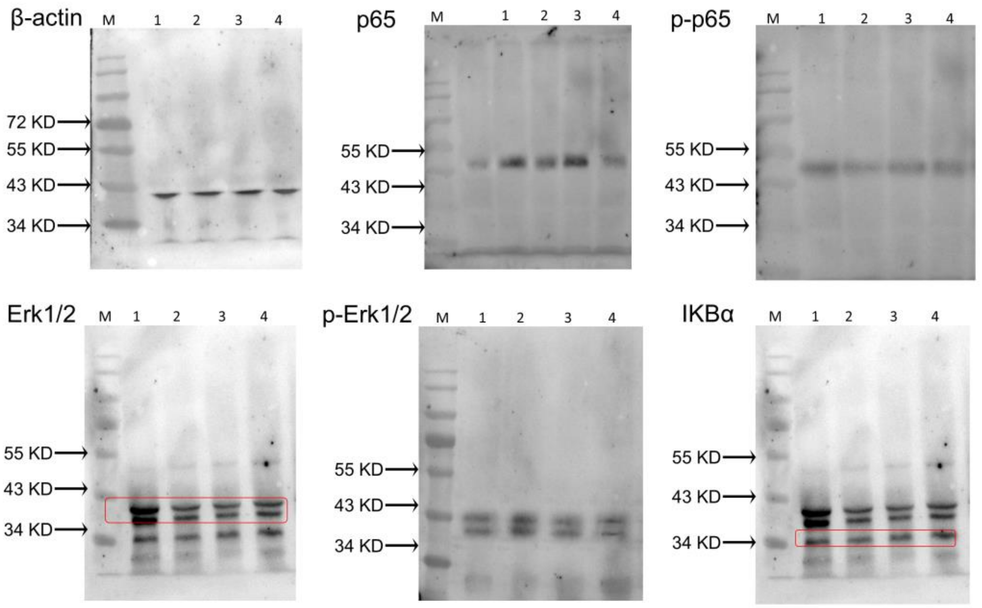

Supplementary Figure 6. **A full and uncropped presentation for Supplementary Figure 5a**. M: protein marker 26617 (Thermo Scientific™), Lane 1: CK, Lane 2: LPS, Lane 3: L7, Lane 4: R7. Three samples from three biological repeats were homogenated and analyzed in a gel electrophoresis.
