# Supplementary material for: A cleavable chimeric peptide with targeting and killing domains enhances LPS neutralization and antibacterial properties against multi-drug resistant E. coli
Source: Commun Biol. 2023 Nov 16;6:1170. doi: 10.1038/s42003-023-05528-0 (PMC10654507; doi:10.1038/s42003-023-05528-0)
Supplement: Supplementary file 3 — Description of Additional Supplementary Files [file 42003_2023_5528_MOESM3_ESM.pdf]

Description of Additional Supplementary Files

File name: Supplementary Data
Description: The source data underlying Figs. 1a–d, 2b, 3b–d, 4a, 5a–g, 6b-c, 7a-f, 8b-c, supplementary Figs. 2b–d, 3a–b, 4, and 5b–d.
